# Age Hardening of Extruded AA 6005A Aluminium Alloy Powders

**DOI:** 10.3390/ma12142316

**Published:** 2019-07-19

**Authors:** Iria Feijoo, Marta Cabeza, Pedro Merino, Gloria Pena, Pilar Rey

**Affiliations:** 1 Department of Materials Engineering, Applied Mechanical and Construction, ENCOMAT Group, University of Vigo, EEI, E36310 Vigo, Spain; 2 AIMEN, Technological Centre, Polígono de Cataboi, E36418 Porriño, Pontevedra, Spain

**Keywords:** aluminium alloy, pre-alloyed powders, powder extrusion, age hardening, microstructural characteristics, mechanical properties

## Abstract

Pre-alloyed micron-sized 6005A Al alloy (AA 6005A) powders, with a Mg/Si atomic ratio of 0.75, obtained by high pressure inert gas atomization were consolidated by uniaxial cold pressing at 200 MPa into cylindrical Al containers and hot extruded at 450, 480 and 500 °C with an extrusion rate of 7:1, followed by artificial T6 precipitation hardening. Ageing conditions were varied between 170 °C and 190 °C and times of 6, 7 and 8 h. The microstructure of the extruded profiles was analysed using X-Ray diffractometry (XRD), light optical microscopy (LOM), scanning electron microscopy (SEM), and transmission electron microscopy (TEM). Differential scanning calorimetry (DSC) was used to study the possible phase transformations. After our results, the peak-aging hardness condition was achieved at 180 °C for 6 h. Mechanical properties of the powder metallurgy (P/M) aluminium alloys consolidated by hot extrusion were superior to those of the extruded profiles of wrought alloy using conventional ingot metallurgy (I/M) billets. AA 6005A wrought P/M alloy via T6 heat treatment shown yield stress of 317 MPa and elongation of 21% at the extrusion pre-heating temperature of 500 °C.

## 1. Introduction

The use of Al alloys in the industry is increasing due to their high strength/weight ratio and reasonable cost. The 6xxx series Al alloys (Al-Mg-Si) are mostly used for the manufacture of extruded products for structural applications especially in construction and transport industries due to their excellent formability, good weldability and corrosion resistance. Among these aluminium alloys, the Al-Mg-Si-(Cu), AA 6005A alloy, highly used in high-speed trains, has an intermediate strength in the 6xxx series, with excellent extrusion performance, good welding properties and corrosion resistance [1,2,3].

Wrought and cast technologies are the most widely used processes for the manufacture of Al alloys. However, in recent years, the interest for powder metallurgy (P/M) technology has increased and it is an alternative to conventional processes. P/M is a technology producing or using metal powder as raw material to produce near net shape components, reducing machining operations and improving performance. In addition, fine grain microstructure and almost unlimited alloy design can be obtained which result in increased corrosion resistance and mechanical properties [4,5,6,7]. Therefore, P/M technology presents very attractive advantages for different industrial sectors with reduction of manufacturing cost. The manufacture of aluminium alloys parts in sectors such as the automotive [8,9,10,11,12] has notably increased in the last decade, becoming the biggest customer of P/M technology.

Among P/M processes, Hot Powder Extrusion (HPE) is a consolidation process for the production of final products from powders, which allows obtaining fully dense materials. In the case of Al alloys, during HPE the oxide layer covering the powder particles breaks down due to the high developed shear stress that allows to obtain a well-bonded microstructure [13]. As it is well known, compacts can undergo microstructural changes during heating up to the extrusion temperature. Therefore, these microstructural changes and their effect on the mechanical properties must be taken into account in the extrusion process. The influence of the extrusion temperature on profiles obtained by conventional ingot metallurgy (I/M) route was widely studied [14,15,16,17,18]. This is not the case for extruded profiles from powders. Therefore, the first objective of this work is to study the influence of extrusion temperature on microstructure and mechanical properties and to determinate the optimum extrusion temperature for manufacturing profiles from AA 6005A powders.

However, after the extrusion process, these alloys show weak mechanical properties that can be improved by suitable heat treatments performed after HPE. Age hardening is the main strengthening mechanism of heat treatable Al alloys, based on the precipitation of very small particles of second phases that improve the hardness and strength of these alloys. In Al-Mg-Si-(Cu) alloys, the main precipitate phases that contribute to the peak-aging hardness are the L, S, C, Q and β″ phases, and alloys with low Cu and high Si contents exhibited higher precipitation hardening than alloys rich in Mg during artificial aging [19,20,21,22,23,24,25].

The influence of the aging heat treatment in extruded profiles of 6xxx series Al alloys obtained by I/M was studied by different authors [21,22,23,24,25]. Nevertheless, the research of extruded profiles from powders of 6xxx series Al alloys submitted heat treatment is very limited [26]. The most investigated P/M aluminium alloys are the 2xxx and 7xxx series [6,27,28,29,30]. Therefore, the second objective of this work is to determine the parameters (temperature and time) of the ageing heat treatment for extruded profiles from AA 6005A powders that provide the alloy with better mechanical properties.

## 2. Materials and Experimental Procedures

The raw material used for this study is a commercially available micron-sized pre-alloyed powder of Al-Mg-Si-Cu with low Cu, AA 6005A, supplied by ECKA Granules Germany GmbH. It was obtained by inert gas atomization of the Al alloy ingot, with a nominal particle size of <63 μm, 99.8% determined by sieve analysis according to ISO 4497:1983. Table 1 shows the elemental chemical analysis given by the manufacturer. As can be seen, the atomic ratio of Mg/Si is 0.75, that is, with Si in excess with respect to the stoichiometric composition Mg_2_Si, therefore the alloy is designated as an “excess-Si”.

The as-received powder was pre-compacted in an Al container at 200 MPa. Extrusion of the powder compacts into the sealed Al metal can was performed at preheating temperatures of 450, 480 and 500 °C, with a rate of 2 mm/min in a horizontal lab-scale computerized extrusion press of 300 tons at the National Center for Metallurgical Research (CENIM) facilities in Spain. The extrusion ratio (ER) was 7:1, which produced a true strain of 1.9. A plane die was used to obtain rectangular section profiles (40 × 3 mm^2^) of approximately 1000 mm length. The profiles were air cooled to room temperature (RT) at the die exit. In order to investigate the age hardening response of the compacted powder extruded profiles, different T6 heat treatments were performed. T6 temper involved a solution heat treatment at 530 °C for one hour in the furnace, and then quenched into iced water. Immediately after, all the specimens for T6 temper were artificially aged at 170, 175, 180, 185 and 190 °C in the oven for varying times 6, 7 and 8 h, followed by air-cooling.

X-ray diffractometry (XRD), thermal analysis, light optical microscopy (LOM), scanning electron microscopy (SEM), transmission electron microscopy (TEM), and hardness and tension testing were employed for the microstructural and mechanical characterization of the extruded alloys. To eliminate the possible effects of microstructural variations between different parts of the extrudates, all the samples were cut from the center of each extruded profile and observed in the direction parallel to the extrusion one. The samples for metallographic study were mounted in a conductive resin, ground and polished with colloidal silica (0.04 μm). The microstructure of the extrudates were studied by LOM, using a Olympus GX-51 microscope (Tokyo, Japan), SEM, using a JEOL-JSM 6510 microscope (Tokyo, Japan) with an accelerating voltage between 0.5 kV to 30 kV equipped with an INCA 400 Oxford Energy-dispersive X-ray spectroscopy analyzer EDS (Oxford, UK), and TEM, using a high resolution transmission electron microscope (HRTEM) JEOL-JEM-2010 FEG model (Tokyo, Japan), operated at 200 kV in bright-field mode. Several samples were etched using 10% NaOH reagent followed by Weck’s reagent (100 mL de H_2_O, 4 g de KMnO_4_ and, 1 g de NaOH) prior to LOM imaging. TEM specimens were prepared with a FIB-SEM FEI Helios NanoLab 400 using the in-situ lift-out method. Quantitative evaluation of grain size was carried out in samples prepared perpendicular to the extrusion direction of the studied specimens. The planimetric method following the ASTM E112 standard applying the AnalySIS auto software 5.0 v incorporated in the Olympus GX-51 was used for that purpose.

XRD was also employed for the qualitative analysis of the phases present in the microstructure. A Siemens model D-5000 diffractometer, with Cu Kα radiation (λ = 1.54056 Å) generated at 40 kV and 30 mA was used for XRD measurement. The scanning range was 2θ = 15–60° with a step size of 0.02° and the counting time 10 s per step. Diffraction peaks were identified using EVA V14 software and the International Centre for Diffraction Data database (ICDD) powder diffraction index.

Differential scanning calorimetry (DSC) was carried out to identify the precipitation reactions on a Mettler Toledo DSC 822 instrument. All DSC scans were realized from RT to 600 °C using a heating rate of 20 °C/min in a nitrogen atmosphere (50 mL/min). Platinum pans were used and pure aluminium (99.99%) was the reference material.

Vickers micro-hardness measurements were performed by using an ENCOTEST DuraScan microhardness tester (Kuchl, Austria) according to UNE-EN-ISO 6507-1:2006, applying a load of 100 gf and a dwell time of 15 s, and the reported hardness value for each material was the average value of 10 indentations. The error intervals were calculated as the standard deviation.

Tensile stress-strain testing was conducted at room temperature using a 250 kN MTS tensile testing machine under an engineering strain rate of 8.3 × 10^−4^·s^−1^, along the extrusion direction. A MTS 25 mm extensometer was used to obtain the axial strain during loading. The yield stress was calculated on a 0.2% offset. The average value of the results of three tensile tests was used for each extrudate. Tensile test specimens were prepared according to standard UNE-EN ISO 6892-1:2017 Appendix D, with 3 mm thickness, 6 mm width, 105 mm total length and 30.36 mm calibrated length. All the specimens were tensioned until failure and surface fracture were studied with a SEM.

## 3. Results and Discussion

### 3.1. Characteristics of as Received AA6005A Powder

**Figure 1:**
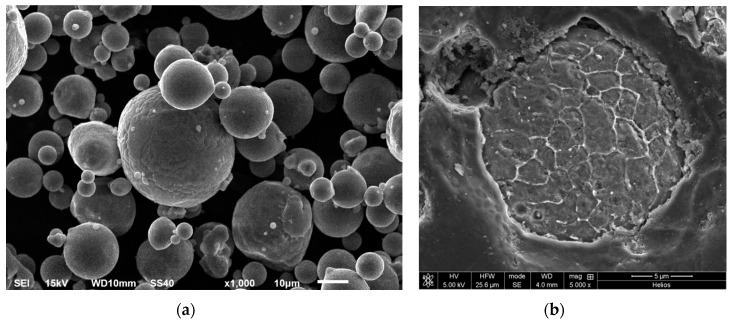
Scanning electron microscopy (SEM) micrograph of AA 6005A powder in the as received condition, (**a**) dispersion in size of the powder particles (**b**) cross section of one particle showing the internal substructure.

Particle size distribution and microstructural analysis of as-received AA 6005A powders were previously reported by the authors [31]. Figure 1 shows the SEM micrograph of powders. The particles are mostly spherically shaped with a mean size of 36.26 µm and dendritic-like morphology at the surface. An internal of the cellular substructure typical of the inert gas atomized powders, was also determined. The average size of the observed internal cells was in the order of 3 µm. The microstructural analysis demonstrated that alloying elements (Si, Mg and Fe) were segregated. In big powders, rod-shaped precipitates of π-phase with Al_9_FeMg_3_Si_5_ composition and very little spherical particles of a ternary composition Al-Fe-Si were analysed along the boundaries of the AA 6005A as the only precipitate second phases. The rapid extraction of thermal energy associated with the inert gas atomization method used to obtain the Al alloy powder, lead to large deviations from the equilibrium producing an extension of the solid solubility limits and the concomitant development of non-equilibrium phases. The small powder particles that had a bigger specific surface area tended to solidify under faster cooling rates, so only elemental segregation was detected. The slower cooling rate of big powder allowed the formation of particles of secondary phases in the boundaries [32].

### 3.2. Characteristics of AA6005A P/M Extruded Alloys

**Figure 2:**
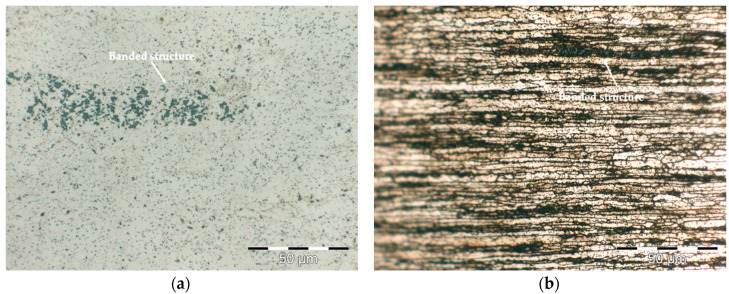

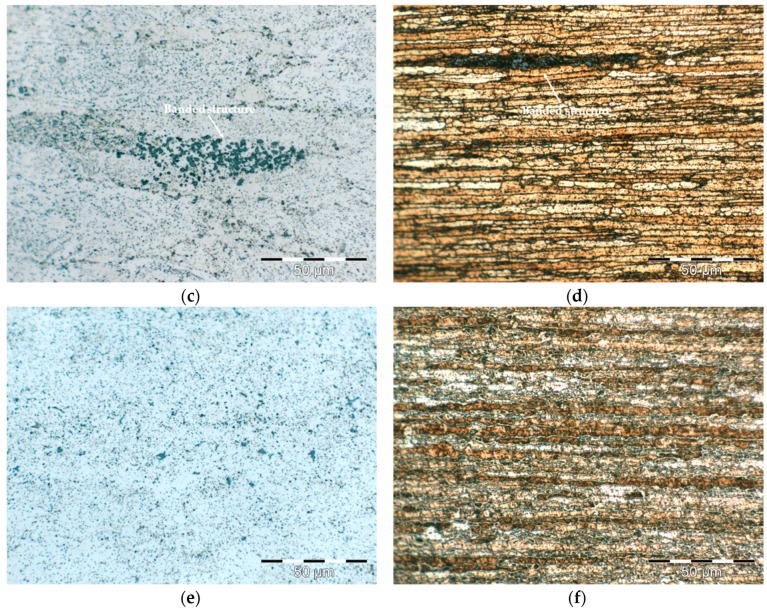
Light optical microscopy (LOM) micrographs of the longitudinal sections showing the microstructure of extruded profiles at preheating temperatures of 450 °C (**a**,**b**), 480 °C (**c**,**d**), and 500 °C (**e**,**f**) from AA 6005A powders. Images on the left: 10% NaOH reagent, images on the right: 10% NaOH and Weck’s reagent.

Figure 2 shows the longitudinal microstructures of the extruded profiles at preheating temperatures of 450, 480 and 500 °C, etched using 10% NaOH reagent (Figure 2a,c,e) and 10% NaOH reagent followed by Weck’s reagent (Figure 2b,d,f). In the microstructural exam of the extruded samples, no oxide particles and no porosity was observed either. Therefore, the used extrusion ratio was sufficiently high to produce full density bulk materials from powder compacts, although the ER used was lower than the values published by other authors [33,34,35]. However, all the specimens exhibited particles of different sizes formed bands parallel to the extrusion direction although the presence of these particle clusters was less evident in the extruded profiles at 500 °C (Figure 2e), which may indicate that at this heating temperature, the quantity of dissolved particles was higher. Banding phenomena normally occurred in the extrusion of Al alloy powders containing segregated phases or in Al based powder mixtures containing components with different hardness and flow stresses that the Al solid solution (Al (α)) matrix [33,34]. In this case, the segregated phases showed different deformation behaviours during extrusion. Softer phases undergo more deformation than the harder phases. As a result, less-deformed phases align with the extrusion direction to form bands.

**Figure 3:**
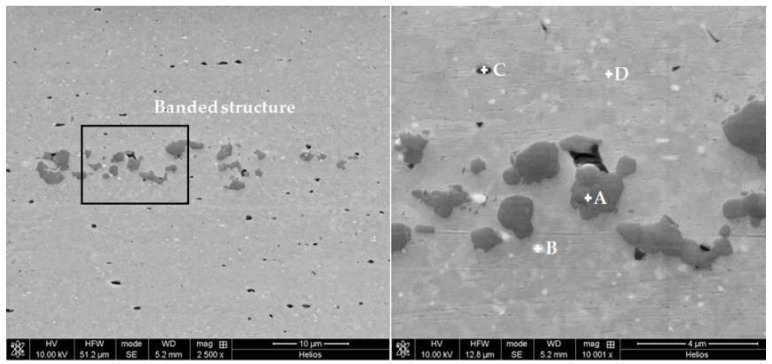
SEM micrographs and EDS analysis of the precipitates of different second phases present in the profiles extruded from AA 6005A powders.

**Figure 4:**
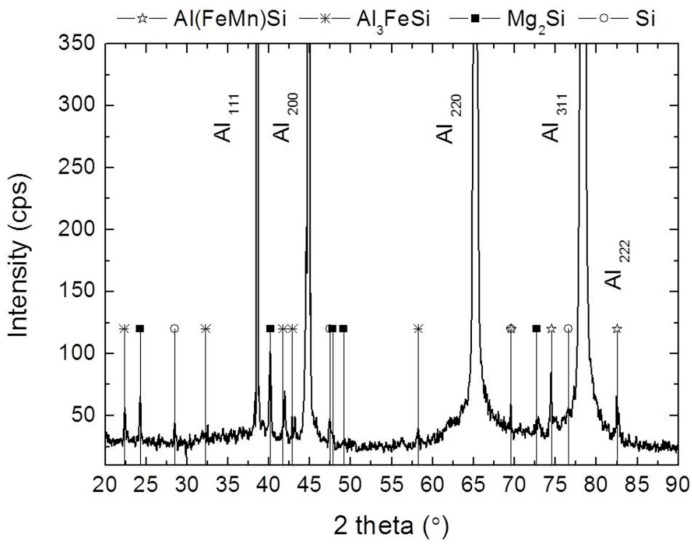
X-Ray diffraction (XRD) pattern of the profiles extruded at 500 °C from AA 6005A compacted powders.

In this case, these elongated bands are mainly composed by grey particles of irregular shape and size up to 3 µm, that are only found in these clusters. As EDS semiquantitative analyses in Figure 3 show, these particles present a high percent of silicon and X-ray diffractograms (Figure 4) which allows them to assign these particles to Si precipitates (ICDD card 27-1402). The presence of this Si dispersoids have been already detected in extruded profiles from Al-0.45Cu-0.5Mg-0.2Si [34]. As they are harder than the Al (α) matrix, its deformation behavior during extrusion favor the alignment with the direction of extrusion.

Other secondary phases present in the banded structures are also found uniformly dispersed in the Al (α) matrix. Thus, the white particles with size down to 1 µm on Figure 3, composed by aluminium, iron and silicon were assigned to Fe bearing intermetallic phase Al_3_FeSi (ICDD card 20-0032). Finally, dark particles less than 1 µm in size containing Mg and Si were also observed (SEM/EDS analysis (Figure 3, spectrum C) and XRD (Figure 4)). The Mg/Si proportion corresponded to the compound Mg_2_Si. The XRD pattern confirms the presence of these precipitates (ICDD card 01-1192).

The longitudinal sections of the specimens on Figure 2 clearly shows the presence of a high fraction of large and coarse grains elongated in the direction of the extrusion, that exhibit a certain internal substructure. In addition, finer grains are present in the microstructure. In order to measure the average grain size of the extruded samples, the planimetric method was applied on five images at 1000× magnification from each one of the extruded profiles, perpendicular to the extrusion direction, analyzing a total area of the 5500 μm^2^ per image. In all cases, the grain size distribution is a uni-modal normal (Gaussian) distribution. The distribution range varies from G = 11 to 17. An important fraction of the grains in included in the larger size ranges, so for 450 °C, 61% of the grains have an average diameter between 8 and 4 μm, while at 480 °C it is the 65%. At 500 °C, this percentage is 66% indicating a slight increase in the overall grain size with increases in the extrusion temperature. The rest of the grains are small grains, under 3, 5 μm, difficult to measure through this method. Differences in average grain size number (G) are almost negligible, being 13.5 for 450 °C and 480 °C, and 13.3 for 500 °C. Then, for the three conditions, extruded profiles are considered fine grained alloys.

SEM images in Figure 3, confirm the heterogeneity in the dimensions of the grains, formed as a result of the interparticle welding during extrusion process [36]. The original spherical shape of the powder particles has been highly deformed by the extrusion leading to very elongated grains in the longitudinal section and slightly more regular in the transversal. The dispersion in grain size visible in these images is minimized in the planimetric method due to de determination at 1000× that hinders the measure of the smallest grains.

**Figure 5:**
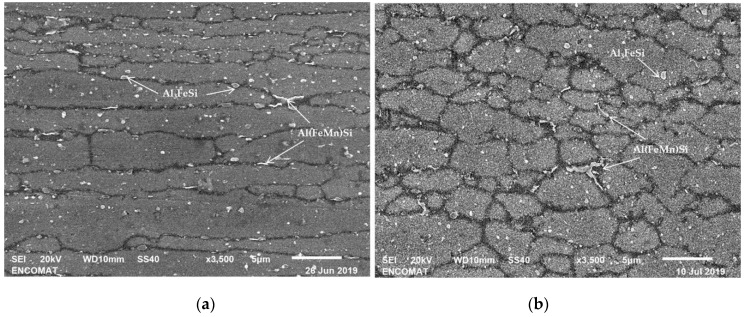
SEM micrographs of the profiles extruded: (**a**) Longitudinal sections and (**b**) transversal sections at extrusion direction.

As it can be observed in Figure 5, after extrusion, the grain boundaries formed from the surfaces of the original powder particles were decorated with precipitates up to 1 µm. The moforlogy of the particles and the semi-quantitative analyses performed by EDS suggested that Al(FeMn)Si particles were present, as confirmed by the X-ray difractrogram in Figure 4. The small precipitates that were initially distributed along the grain boundaries of the internal structure of the AA 6005 powder particles were dispersed in the middle of the grains, Al_3_FeSi, Mg_2_Si.

From the point of view of microstructural development, dynamic recovery (DRV) and/or dynamic recrystallization (DRX) can take place during hot deformation. In the case of metallic materials such as Al with high stacking fault energy (SFE), DRV, which involves development of a sub-grain structure within elongated grains, is energetically more favorable, inhibiting discontinuous dynamic recrystallization (DDRX), which operates by nucleation and grain growth. Therefore, DDRX operates in low to medium SFE materials at high temperatures (T > 0.5 Tm). However, the formation of new grains during hot deformation of Al alloys has been many times reported by continuous dynamic recrystallization (CDRX). This process involves the transformation of low angle grain boundaries (LAGBs) to high angle grain boundaries (HAGBs), and geometric dynamic recrystallization (GDRX), generated by the fragmentation of the initial grains when the Al is deformed to large strains at elevated temperatures [35,37,38,39,40].

CDRX takes place when the grain boundaries are pinned by small particles, such as precipitates and occurs due to the progressive accumulation of dislocations in LAGBs, which leads to an increase in misorientation until the critical value θc (θc ≈ 15°) is reached and the transformation of LAGBs into HAGBs is produced. It was found that this DRX process takes place in all metals and alloys irrespective of their SFE when the deformation temperature is relatively low (T < 0.5 Tm), by severe plastic deformation. At high temperatures, however, CDRX is most frequently observed in high SFE materials [36].

In the present case, the main part of the structure consists of large elongated grains along the extrusion direction with equiaxial sub-grain structure, as result of a DRV process, as shown in SEM images in Figure 5. However, the presence of dispersoids (Figure 2) and second phase particles with size > 1 μm (Figure 5) can also play an important role on the kinetic of the recrystallization process. According to K. Huang et al. [38] and R. D. Doherty et al. [39], during the hot extrusion process, local deformation around these particles is greater than the deformation produced in the Al (α) matrix without particles, creating a region of high dislocation density and increasing the susceptibility of nucleation of new grains [33,34,37]. Therefore, the establishment of the CDRX mechanism can explain the existence of the small recrystallized grains present in the microstructure.

The profiles extruded at 500 °C (Figure 2e,f) presented a lower number of bands, resulting in a more homogeneous and controlled grain size distribution.

The average Vickers microhardness values of the specimens extruded at 450, 480 and 500 °C were 48 ± 1, 46 ± 4 and 48 ± 1 HV 0.1, respectively. This indicates that the extruded P/M billets revealed no significant dependence of hardness on the extrusion temperature within the considered range, as could be expected given the similarity of their structures.

On the basis of the above the temperature of 500 °C, this was considered to be more adequate for the hot extrusion of AA 6005A compacted powders and, consequently, the selection of the age hardening (T6 temper) parameters will be carried out on the profiles extruded at this preheating temperature.

### 3.3. Age Hardening

The precipitation events in 6xxx alloys are quite complex and highly dependent on the proportion of the alloying elements. So, in quaternary Al-Mg-Si-Cu alloys, the phases and their sequence during precipitation are different if considering balanced (Mg_2_Si stoichiometry), excess-Si (wt % Mg < 1.732* wt % Si) and high or low Cu level compositions.

In excess-Si + low Cu Al-Mg-Si-(Cu) alloys the following precipitation sequence has been proved to take place [25,41,42]:

Supersaturated solid solution (SSS) → atomic clusters → coherent Guinier-Preston (GP) zones → needle-shaped β″ precipitates → rod-shaped β’ precipitates → plates equilibrium β (Mg_2_Si) precipitates, Si.

**Figure 6:**
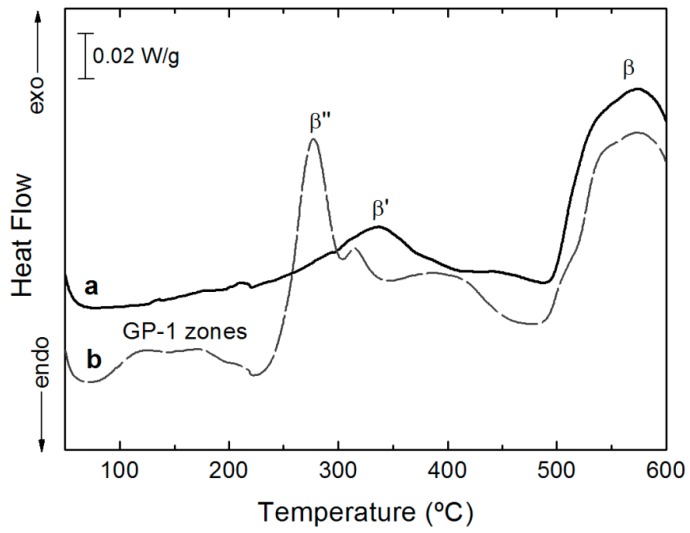
Differential scanning calorimetry (DSC) curves of the AA 6005A powders extruded at 500 °C from AA 6005A compacted powders solution treated at 530 °C and quenched a and untreated b.

In Figure 6, the DSC curve of the AA6005A powder extruded at 500 °C, obtained after solution treatment, was compared with the DSC scan of the untreated specimen extruded in the same conditions. The number of peaks and their positions in the DSC scan of the heat-treated specimen are similar to those reported by Dutta and Allen [39] and Edwards et al. [40], although the peaks are shifted to higher temperatures in the present work. This was most likely due to the faster heating rate used in the present work (20 °C/min, cf. 10 °C/min for Dutta and Allen and 5 °C/min for Edwards et al.). Several differences are noted between DSC scans of heat-treated and untreated specimens. In the scan of heat-treated specimen, the exothermic peaks in the temperature interval between 100–200 °C were linked to the formation of the very fine, fully coherent with the Al (α) matrix GP zones and/or cluster formation [19,20,25]. These peaks were not observed in the untreated specimen. Another exothermic peak was detected at 275 °C, associated with the precipitation of semi-coherent metastable β″phase with needle-shaped morphology, one of the strengthening phases in the Al-Si-Mg-(Cu) alloys [25]. The transformation of metastable β″phase to the semi-coherent rod-like β’phase and lath-like precipitates produced the following exothermic peak centered at 320 °C. In the untreated specimen, a broader peak of the semi-coherent β’phase formation was observed at 340 °C, indicating a precipitation delay and a higher volume of precipitated particles. The last exothermic peak at approximately 575 °C for both specimens was linked with the precipitation of the equilibrium β phase (Mg_2_Si) as plates [19,25].

The results derived from DSC curves were in agreement with the measured Vickers microhardness values. The untreated specimens present an average hardness of 48 HV 0.1, lower than the obtained in heat-treated specimens (52 HV 0.1). The precipitation after the extrusion process of the phases incoherent with Al (α) matrix did not produce any hardening in the alloy. However, in the heat-treated extruded profiles, a slight increase in the average hardness was measured due to the distortion created in the Al lattice supersaturated by the alloying elements, even when the values were low.

### 3.4. Precipitation Hardening of the Extruded Profiles

Table 2 summarizes the effect of ageing time and temperature on Vickers microhardness of compacted powders extruded at 500 °C and solution treated. Microhardness values in black color and red color in the table indicates regions with different hardness values: The black colour corresponds to heat treatment conditions producing hardness very similar to each other, between 102 and 109 HV 0.1, close to an average value of 105 HV 0.1; whereas the dark grey cells show the conditions leading to values lower than 100 HV 0.1. These results also confirm the higher hardness of the aged specimens compared to those after solution treatment. Following these results, peak aging conditions are 180 °C for 6 h, with a hardness value of (109 HV 0.1), and the minimum hardness (71 HV 0.1) was produced after treatment at 190 °C for 8 h.

Compared to these results, the measured peak hardness in extruded profiles of the same alloy manufactured by conventional I/M route is 110 HV 0.1 after aging at 175 °C for 8 h, was very similar to the hardness achieved in the specimens of AA6005A compacted powder extruded at 500 °C and age hardened at 180 °C for 6 h. Therefore, by increasing aging temperature by 5 °C the aging time can be reduced by 2 h with respect to the age hardening of the conventional alloy, which implies a reduction of the production costs.

**Figure 7:**
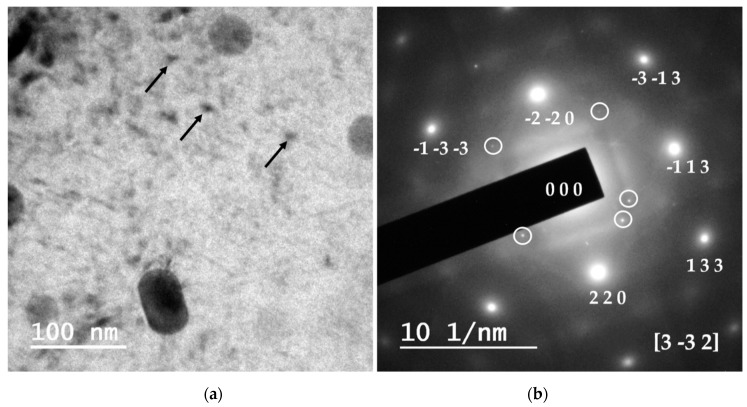
A transmission electron microscopy (TEM) bright-field image (**a**) and selected area diffraction (SAD) pattern (**b**) of an extruded profile at 500 °C from compacted AA 6005A powders specimen aged at 180 °C for 6 h (peak-aging hardness condition).

Figure 7 shows the corresponding TEM bright-field image and selected area diffraction pattern (SADP) of a specimen of the profile extruded at 500 °C from compacted AA 6005A powders and aged at 180 °C for 6 h (peak hardness). The embedded β″ precipitates present some obvious Lobe-shaped coherency strain-field contrast due to are aligned with the long axis parallel to the electron beam, as shown by the solid arrows. This orientation has very low diffraction effect. These precipitates are semi-coherent and share planes with the Al (α) matrix [19,20]. The corresponding SADP under the [$3\;\bar{3}\;2$]_Al_ zone axis show in addition to the f.c.c. crystal lattice of aluminium in this zone axis, show very weak diffraction spots (indicated by the circle in the image) that could correspond at typical “cross shaped” diffraction streaks arising from the metastable β″. The SADP depends on the precipitate shape, their orientation and order of the diffraction spots. W. Yang et al. [43] describes that the high-order diffraction spots produce the “cross shaped” streaks, and the elongated streaks around the high-order diffraction spots are produced by the shape-effect of precipitates.

**Figure 8:**
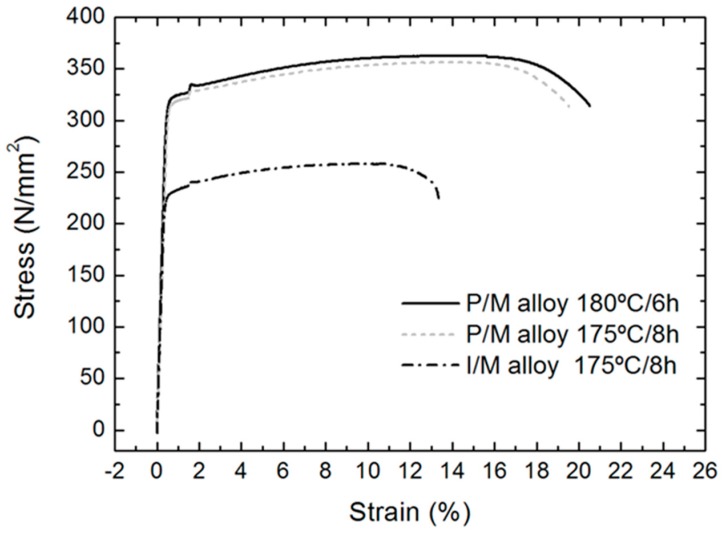
Tensile engineering stress–strain curves of aged extruded profiles at 500 °C from AA 6005A compacted powders (AA 6005A powder metallurgy (P/M) alloy) and from the ingot metallurgy (I/M) route (AA 6005A I/M alloy).

In an attempt to differentiate the effect of the initial processing of the alloy from that produced by the aging heat treatment, the tensile behaviour of samples extracted from extruded profiles of conventional AA 6005A I/M alloy aged hardened at 175 °C for 8 h was compared to that of samples from compacted powders extruded at 500 °C (P/M) and age hardened in two different ways: The same conditions that the I/M samples displayed and the peak hardness condition. Figure 8 shows the obtained tensile curves and Table 3 summarizes the resulting mechanical properties.

As it can be clearly seen, the performance in the tensile test of both P/M alloys was very similar. AA 6005A P/M specimens in the peak hardness presented slightly higher values of yield strength, tensile strength and maximum elongation, than the samples heat treated in the other condition. But, on the contrary, an important difference between conventional ingot metallurgy profiles (I/M) and those prepared by powder metallurgy, being both extruded at 500 °C and age hardened at 175 °C for 8 h. According to these results, an increase of ~40% in yield strength and ultimate strength and of ~47% in strain to failure elongation.

The superior strength of the P/M alloys can be easily attributed to the finer grain microstructure, derived from fine dimension of the initial micron-sized AA 6005A powders. In terms of the Hall-Petch effect of grain size strengthening, grain boundaries can act as barriers to the motion of dislocations, limiting plastic deformation at room temperature. The drawback of this approach is the reduction in ductility, so the parallel increase of elongation is not evident.

Recent studies have shown that simultaneous improvement in strength and ductility of bulk alloys is possible on ultrafine-grained (UFG) or nanocrystalline alloys, if their microstructure allow the delocalization of strain concentration promoting extended distribution of plastic flow [44,45]. This effect can be accomplished creating heterogeneous nanostructures like a bimodal distribution of grain size, gradient structures, nanotwins or precipitation-hardening [45].

In the case of age hardening aluminium alloys, the published results [45,46] show that the presence of some undissolved second-phase particles may contribute to dislocation accumulation and grain refinement during deformation stage. As a consequence, an important increase in precipitation of nano-sized coherent or semi-coherent particles is produced during the process of aging producing leading to an improvement in strength via resistance to dislocation slip and trapping. The study of S. Cheng and cols. [46] has demonstrated that for promoting the strength-ductility synergy it is necessary to achieve high density of nanoscale second-phase precipitates as well as low dislocation density are desirable. Additionally, high-temperature and short-time aging are shown to be most efficient in simultaneous enhancement of strength and ductility.

The results of the present work can be explained according to the above arguments. During hot extrusion an ultrafine microstructure was formed as a consequence of the plastic deformation and the recrystallization processes in which the presence of non-solved second phase particles plays an important role, as it was previously explained. The high density of dislocations generated during deformation is mostly disappeared due to recovery and recrystallization. After T6 treatment a high density of nano-sized β″ precipitates, is produced. This process gives rise to a heterogeneous nanostructure in which the extremely low dislocation density in the matrix enhances deformation and allows dislocation accumulation before saturation during tensile test, increasing the work-hardening rate. The nano-sized β″ provides effective sites for trapping and piling-up dislocations, increasing also the work-hardening rate and the ultimate strength of the alloy, concurrently achieving improved strength and ductility in the AA 6005A P/M alloy.

**Figure 9:**
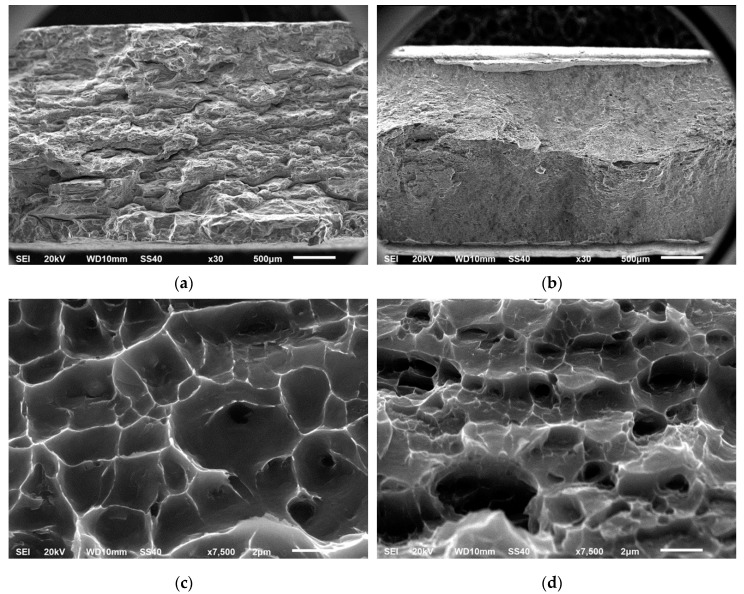
Fracture surface topography of AA 6005A specimens extruded at 500 °C treated in the peak-aging hardness condition after tensile tests. SEM macrographs of (**a**) I/M route and (**b**) P/M route, SEM micrographs of (**c**) I/M route and (**d**) P/M route.

Figure 9 provides the SEM micrographs of the fracture surfaces after tensile testing at room temperature, illustrating the differences between the AA 6005A I/M (a, c) and P/M specimens (b, d). Macrograph in Figure 9a evidences the lower plastic deformation before fracture of the conventional alloy compared to that of the P/M alloy in Figure 9b, which shows the characteristic "necking" consequence of the extensive elongation and the shear surfaces angled at 45° to the applied load, with rough and fibrous appearance.

Micrographs in Figure 9c,d show dimples on fracture surfaces of both specimens, representative of the ductile fractures and derived from the slow nucleation, growth and coalescence of micro-voids. Their size is determined by the density and distribution of sites in which microvoids can nucleate, that is, stress concentrators as second-phase particles and grain boundaries. Dimple shape is influenced by the state of stress and the deformation leading to fracture; therefore, deeper dimples are normally associated with higher ductility, while lower ductility leads to shallower dimples [47].

The fracture surfaces of the extruded AA 6005A P/M alloy aged in the peak-aging hardness condition reveals a less uniform pattern of finer and deeper dimples than the conventional I/M alloy, consistent with its lower grain size and higher ductility.

## 4. Conclusions

In this study, pre-alloyed micron-sized powders of AA 6005A alloy, obtained by high-pressure inert gas atomization, were consolidated by pre-compaction and hot extrusion to obtain flat bar profiles that were finally strengthened by precipitation hardening (T6). The extrusion pre-heating temperature and T6 heat treatment conditions were investigated and selected to obtain the best mechanical properties.

The mechanical properties of these P/M alloys measured in tensile tests were compared to those from conventional AA 6005A I/M alloy extruded profiles. From the obtained results, it can be concluded that:

- The temperature selected temperature for hot extrusion was 500 °C, because the banded structure due to the presence of segregated phases in the pre-alloyed powders is less visible and a more uniform grain size distribution was obtained.
- Peak hardening conditions (T6) for the precipitation hardening of the P/M alloy were: 180 °C and 6 h. Temperature was slightly higher and the time was shorter than those normally used in the conventional route, in good agreement with the published results indicating that high-temperature and short-time aging are more efficient to produce a simultaneous enhancement of strength and ductility.
- The extruded profiles produced by powder metallurgy, hot extruded and aged at peak hardness conditions presented superior mechanical properties than the extruded profiles from conventional ingot metallurgy, achieving a simultaneous improvement of ~40% in strength and ~47% in ductility.
The increase in both properties can be explained by combined effect of a UFG structure and a high density of nano-sized β″ precipitates.
This enhancement in properties can open the doors for the use of components extruded from AA 6005A P/M aerospace or military industries where the cost of manufacturing powders can be compensated with best performance of the alloy.

## Figures and Tables

**Table:** 

Spectrum	Element at (%)			
	Al	Mg	Si	Fe
A	58.30	0.40	41.09	0.21
B	78.81	0.57	17.84	2.78
C	77.26	15.54	7.20	-
D	98.84	0.68	0.46	0.03

**Table 1:** Chemical composition of AA 6005A powder in the as-received condition (at %).

Si	Fe	Cu	Mn	Mg	Cr	Zn	Ti	Mn + Cr	Al	Oxygen (ISO 4491/4)
0.845	0.087	0.047	0.064	0.635	0.005	0.045	0.056	0.069	Bal.	0.118

**Table 2:** HV 0.1 hardness obtained in profiles from AA 6005A powders extruded at 500 °C and age hardened at different temperatures and time.

Time (h)	Temperature (°C)				
	170	175	180	185	190
**6**	104 ± 2	103 ± 1	109 ± 1	95 ± 1	96 ± 1
**7**	105 ± 1	106 ± 3	106 ± 7	99 ± 3	86 ± 5
**8**	102 ± 1	106 ± 2	101 ± 1	99 ± 4	71 ± 5

**Table 3:** Mechanical properties of age hardening extruded profiles at 500 °C from AA 6005A compacted powders (AA 6005A P/M alloy) and from I/M route (AA 6005A I/M alloy).

Alloy	Aging Treatment		Mechanical Properties		
	Time (h)	Temperature (°C)	0, 2%YS (MPa)	UTS (MPa)	Elongation (%)
AA 6005A P/M alloy	6	180	318 ± 2	359 ± 3	21.33 ± 0.76
AA 6005A P/M alloy	8	175	313 ± 3	354 ± 4	19.33 ± 1.75
AA 6005A I/M alloy	8	175	228 ± 2	257 ± 1	14.50 ± 1.00
